# Epilepsy. Oxide of Zinc

**Published:** 1854-12

**Authors:** 


Epilepsy. Oxide of Zinc.—The oxide of zinc, so strongly recommended by Herpin in epilepsy has been tried both by Moreau and Delasiauve. Moreau experimented on eleven patients, and rigorously observed Herpin's instructions, but the results were completely negative. Delasiauve's experience, of a still larger scale, is to the same effect. In reference to the employment of the oxide of zinc, we may mention the interesting observations of Michaelis, who, in experiments on animals, found the zinc in the liver, bile, blood, spleen, lungs, heart, brain, and urine. The oxide appears to be dissolved by the lactic acid in the stomach; it should, therefore, not be combined with magnesia, which would neutralize the acid.—*Traité de l'Epilepsie.*

